# A Case of Lower-Pole Moiety Ureteral Stenosis Close to Ureteropelvic Junction in an Incomplete Duplicated Collecting System Managed by Retrograde Balloon Dilatation that Needed a 2nd Ureteral Stent to the Upper-Pole

**DOI:** 10.1155/2020/8835213

**Published:** 2020-07-16

**Authors:** Seiichi Saito

**Affiliations:** Art Park Urology Hospital & Clinic, Ishiyama-Higashi 3-1-31, Minami-ku, Sapporo 005-0850, Japan

## Abstract

A 71-year-old woman presented at our institution with the chief complaints of left back pain and fatigue. Radiographic examination revealed left ureteral stenosis close to ureteropelvic junction of the lower-pole with a left incomplete duplicated collecting system. Transurethral retrograde balloon dilatation under general anesthesia was performed, and a ureteral stent was inserted to the lower-pole; however, there was urinary leakage from the upper-pole at the dilated ureteral stenosis lesion, and therefore, another ureteral stent was inserted to the upper-pole just after the first stent insertion. Both stents were removed at 6 weeks and subsequent intravenous pyelography confirmed resolution of the obstruction. The patient has remained asymptomatic during 2 years of follow-up.

## 1. Introduction

The ureteropelvic junction (UPJ) of the lower-pole, both with a complete and incomplete duplicated system, is a common cause of obstruction. Although minimally invasive treatment like a retrograde endoscopic approach has been reported, most cases are successfully managed [1–4]. I herein report a case with ureteral stenosis close to UPJ of the lower-pole in an incomplete duplicated collecting system managed by retrograde balloon dilatation. However, there was urinary leakage from the upper-pole at the dilated ureteral stenosis lesion, and therefore, another ureteral stent needed to be inserted to the upper-pole.

## 2. Case Presentation

**Figure 1:**
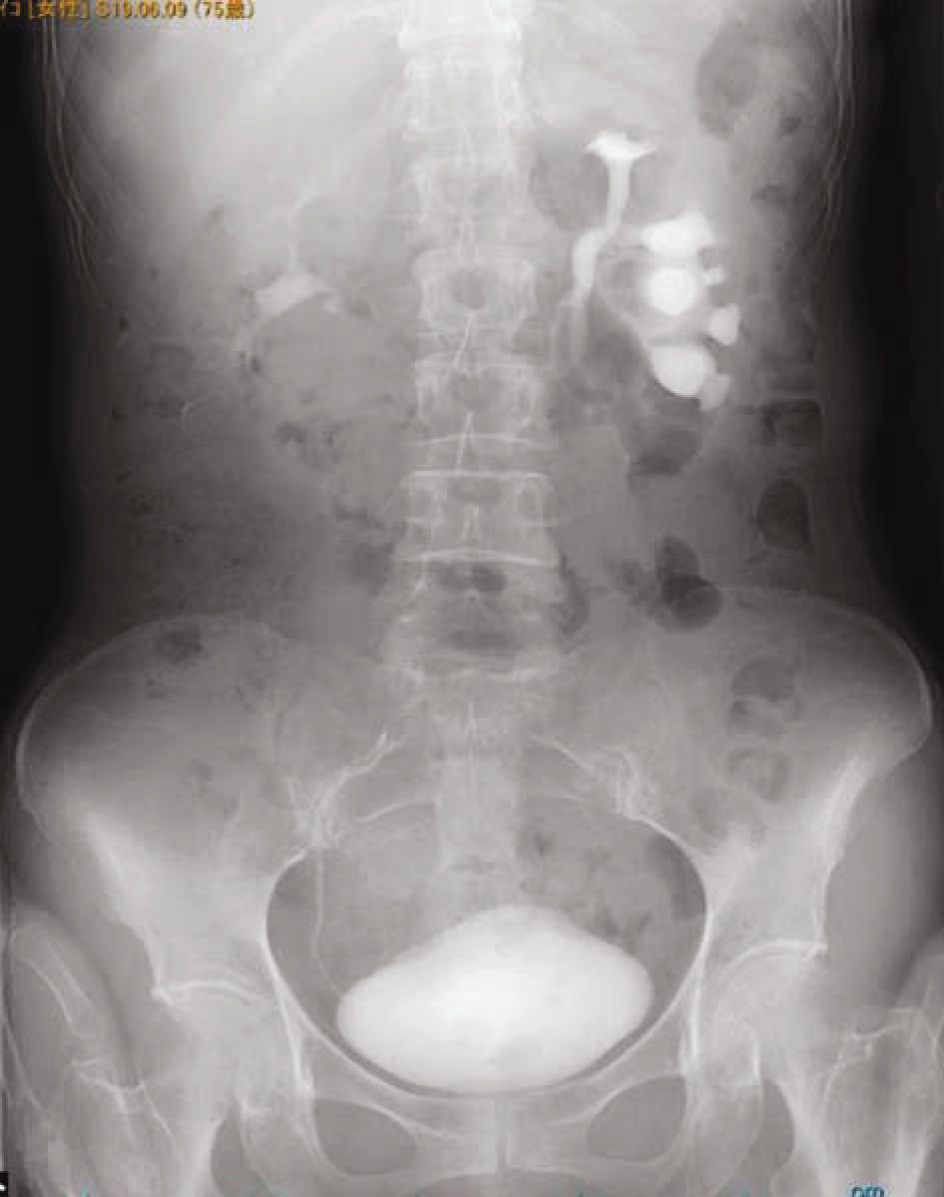
IVP shows hydronephrosis of the left lower-pole in the incomplete duplicated collecting system.

**Figure 2:**
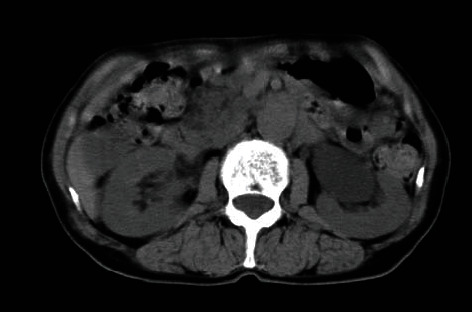
CT shows hydronephrosis of the left lower-pole.

**Figure 3:**
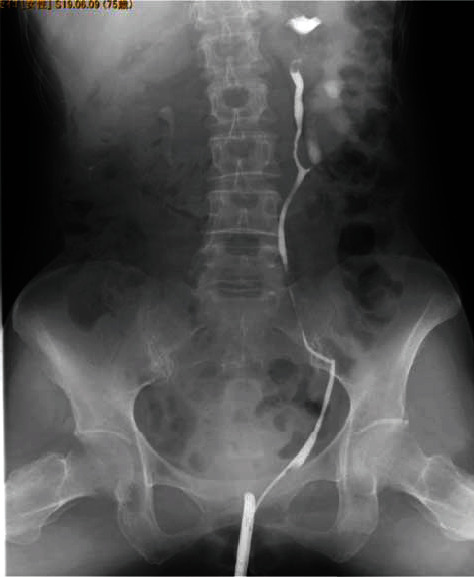
RP reveals UPJO of the left lower-pole in the incomplete duplicated system.

**Figure 4:**
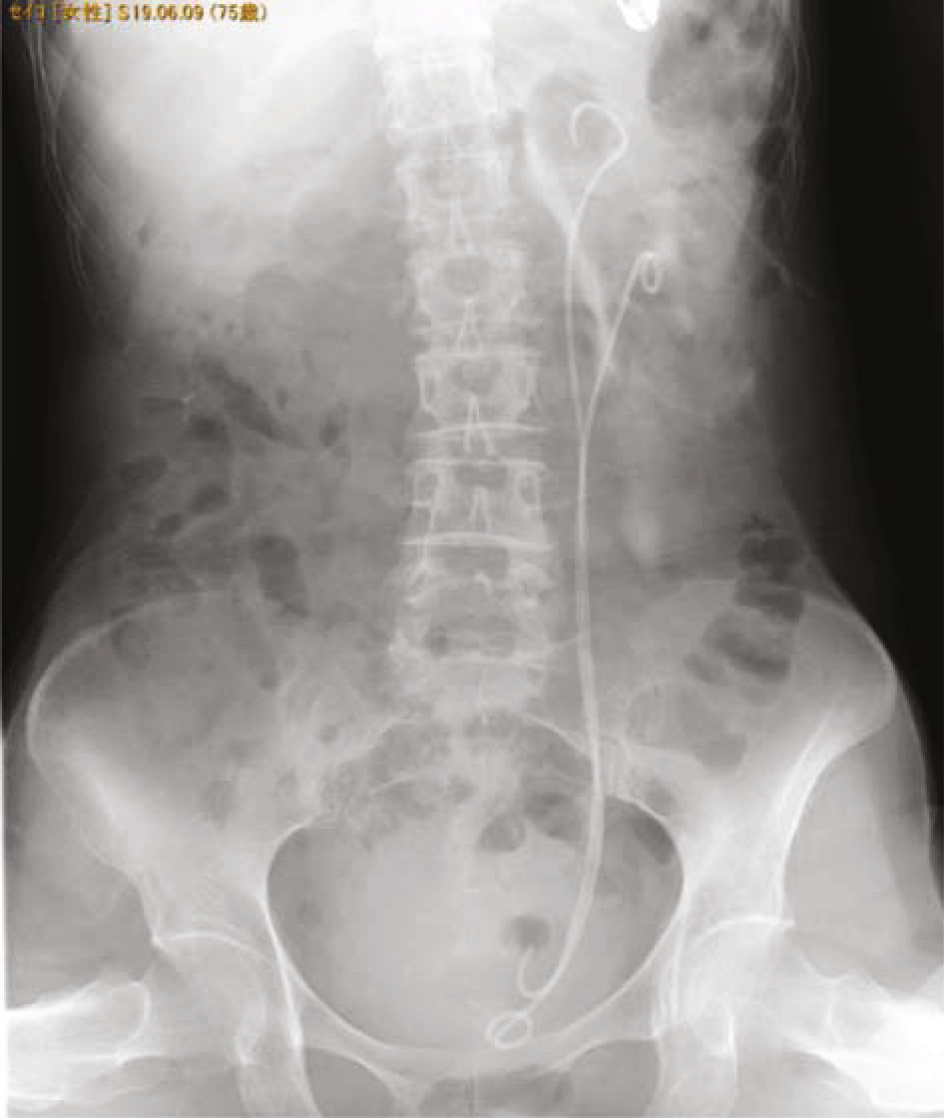
Contrast leakage from the upper-pole of the incomplete duplicated collecting system after the inflation of the UPJO, and stents were placed to the upper-pole and lower-pole.

**Figure 5:**
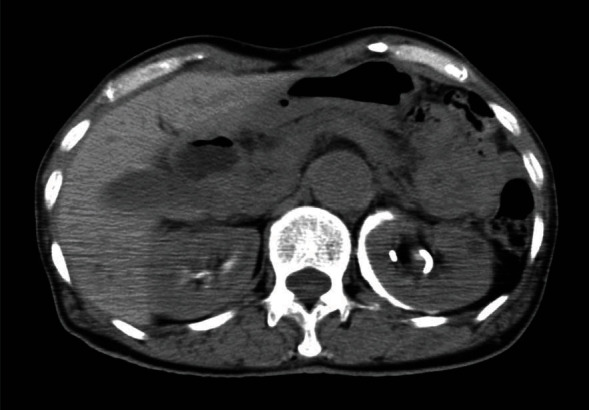
CT shows contrast leakage in the retroperitoneal space.

A 71-year-old woman was referred with dull left back pain and fatigue for 1 year. The patient was evaluated by abdominal computed tomography (CT) and intravenous pyelography (IVP), which revealed incomplete duplication of the left urinary system with lower pole moiety hydronephrosis (Figures 1 and 2). Retrograde pyelography (RP) showed ureteral stenosis close to UPJ with severe hydronephrosis of the lower pole moiety (Figure 3). With the patient in the dorsal lithotomy position under general anesthesia, a 0.038-inch guidewire was inserted into the lower pole of the collecting system, and balloon dilatation (X-Force, 6 mm × 4 cm, Bard Urological Division, C. R. Bard Inc. Covington, GA, 30209, USA) was performed at the narrowed ureteral lesion. Finally, a ureteral stent (InLay Optima, 6Fr × 22 cm, Bard Medical Division, C. R. Bard Inc. Covington, GA, 30209, USA) was inserted to the lower-pole of the collecting system. However, there was urinary leakage with contrast from the upper-pole at the dilated ureteral stenosis lesion, and another ureteral stent (same size and same company) was inserted to the upper-pole of the collecting system (Figures 4 and 5) just after the balloon dilatation. This procedure lasted for 115 minutes, and the patient stayed in the hospital for 5 days. The patient was asymptomatic during the six weeks in which she had two 6 Fr stents in her left ureter. And she was on antibiotics while stenting. Both stents were removed at 6 weeks after operation.

**Figure 6:**
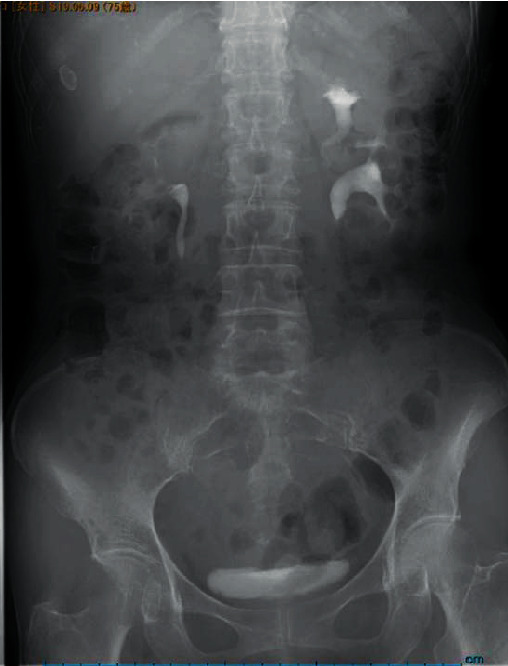
IVP shows the improvement of the hydronephrosis of the left lower-pole 3 months postoperatively.

An IVP at 3 months postoperatively showed reduction of the hydronephrosis in the lower-pole moiety (Figure 6), and the patient remained asymptomatic during 2 years of follow-up.

## 3. Discussion

Duplication of the renal collecting system is the most common upper urinary tract anomaly, occurring in 0.5% of the nonselected population [5]. Of these duplicated systems, approximately 70% are incomplete [6]. Ureteropelvic junction obstruction (UPJO) is the most common cause of pyelocaliectasis, which occurs in approximately 1 in 1500 births [7]. In complete and incomplete duplex systems, UPJO of the lower-pole is a common cause of obstruction [2]. This may be explained by the fact that the lower segment is anatomically the analogue of a single renal system, which usually corresponds to about two-thirds of the parenchyma, and at least 2 calyces and a true renal pelvis [2].

Several endoscopic techniques have been developed as alternatives to open surgery, and the endoscopic approach to UPJ has been successful for both antegrade and retrograde procedures, offering the advantages of shorter operative time, less morbidity, reduction of postoperative analgesic requirements, and shorter hospital stay. Retrograde endoscopic procedures thought to be the most minimally invasive, endopyelotomy, laser incision, Acucise, and balloon dilatation have been reported to be performed successfully.

In this case, transurethral retrograde balloon dilatation was performed for the ureteral obstruction close to UPJ of the lower-pole with a left incomplete duplicated collecting system, after which a ureteral stent was inserted to the lower-pole. However, there was urinary leakage at the dilated ureteral stenosis lesion, and therefore, another ureteral stent needed to be inserted to the upper-pole because the ureteral obstruction was close to the junction of upper and lower poles in the incomplete duplicated collecting system; therefore, urinary leakage started from the operated ureteral stricture lesion from the upper-pole. Fortunately, a 2nd ureteral stent could be inserted to the upper-pole just after balloon dilatation and urinary leakage was minimal after operation. Although laparoscopic or open approach might be safer and effective from the beginning in this case, retrograde endoscopic procedures were thought to be the most minimally invasive and should be tried first.

## 4. Conclusion

When we manage UPJO and/or ureteral stenosis close to UPJ of the lower-pole with an incomplete duplicated system by retrograde balloon dilatation, we should keep in mind the possibility of urinary leakage from the upper-pole and the need for another stent to the upper-pole.

## Abbreviations

UPJ: Ureteropelvic junction
UPJO: Ureteropelvic junction obstruction
CT: Computed tomography
IVP: Intravenous pyelography
RP: Retrograde pyelography.
